# Supplementary material for: Integration of Whole-Genome Resequencing and Transcriptome Sequencing Reveals Candidate Genes in High Glossiness of Eggshell
Source: Animals (Basel). 2024 Apr 9;14(8):1141. doi: 10.3390/ani14081141 (PMC11047648; doi:10.3390/ani14081141)
Supplement: Supplementary file 1 [file animals-14-01141-s001.zip › animals-2923695-supplementary.pdf]

Supplementary materials

Table S1. Specific primers used for qPCR validation

Gene	Primer	Sequence (5'-3')	Size
MRC1	Forward	GGAGGCCACTACTCTAAGCCA	21
	Reverse	GCACTGCGTTTTGTGTTGAG	20
INPPL1	Forward	GACAGTGAGTCCATCGCAGG	20
	Reverse	AAGCACTTGGCCTGGATACC	20
C1S	Forward	AGACACTGGTGGACGTAAGG	20
	Reverse	GCTGCATTAGCCCAGACAGG	20
TRHR	Forward	TCAGGAGCAAGGACAGCAAT	20
	Reverse	CACGGGACTGAGCGGATACA	20
ASB12	Forward	ACGTCTGCTTTGCCTGTCTT	20
	Reverse	CTGGCTGCATGGGTGAAAAA	20
CRISP3	Forward	ATGTGTGCCATTACTGCCCT	20
	Reverse	ACTTGCAAGGATTGGTGCAT	20
DUSP5	Forward	GGCAGACATCAGCTCACACT	20
	Reverse	CCATGCAGATGGTGGGAGAG	20
EOMES	Forward	TCCCCGAAACGCAGTTCATA	20
	Reverse	CAGGGACAATCTGGTGGGAT	20
MSMO1	Forward	GTTCTTCTGTGGGCATGGGT	20
	Reverse	ACGGGCTCCAGCATAGAAAG	20
CRMP1	Forward	ACAAAAGCGCATCCTGGAGA	20
	Reverse	TGGTGATGGCACGGAAAACT	20

Table S2. Glossiness values of experimental units used for sequencing (*n*=5)

<table>
<tr><th>Experimental unit</th><th>Gloss</th><th>mean value</th><th>SD</th><th>P-value</th><th>between-group variance</th><th>within-group variance</th></tr>
<tr><td>E4-3</td><td>2.05</td><td rowspan="5">2.23</td><td rowspan="5">0.144</td><td rowspan="10">1.04E-05</td><td rowspan="5">0.0166</td><td rowspan="10">5.715</td></tr>
<tr><td>E4-8</td><td>2.15</td></tr>
<tr><td>E4-4</td><td>2.2</td></tr>
<tr><td>E4-5</td><td>2.35</td></tr>
<tr><td>E4-10</td><td>2.4</td></tr>
<tr><td>E3-11</td><td>4.55</td><td rowspan="5">5.12</td><td rowspan="5">0.649</td><td rowspan="5">0.3366</td></tr>
<tr><td>E3-9</td><td>4.7</td></tr>
<tr><td>E3-1</td><td>4.7</td></tr>
<tr><td>E3-3</td><td>5.75</td></tr>
<tr><td>E3-8</td><td>5.9</td></tr>
</table>

Table S3. Quality analyses of transcriptome sequencing

Experimental unit	Reads No.	Bases (bp)	Q30 (bp)	Q20(%)	Q30 (%)
E4-3	41050906	6198686806	5766204771	97.29	93.02
E4-8	45198208	6824929408	6319419747	97.09	92.59
E4-4	46971730	7092731230	6604519365	97.35	93.11
E4-5	43292752	6537205552	6098667398	97.37	93.29
E4-10	42440424	6408504024	5966018352	97.34	93.09
E3-11	44938098	6785652798	6350066200	97.52	93.58
E3-9	46156182	6969583482	6480337698	97.28	92.98
E3-1	43566000	6578466000	6111495303	97.22	92.9
E3-3	39757262	6003346562	5575300130	97.25	92.86
E3-8	50883826	7683457726	7148938478	97.33	93.04

Table S4. Quality analyses of whole genome resequencing

Experimental unit	HQ_Reads	HQ_Reads(%)	Reads_Num	Total_Bases(bp)	GC(%)	Q20(%)	Q30(%)
E3-3	228,621,120	95.3	239,888,126	36,223,107,026	43.24	96.73	91.82
E3-8	250,403,102	94.92	263,811,314	39,835,508,414	44.08	96.56	91.57
E3-9	283,568,170	92.9	305,237,280	46,090,829,280	51.92	95.68	90.3
E3-1	224,933,686	93.06	241,718,326	36,499,467,226	47.88	95.91	90.98
E3-11	260,753,256	95.11	274,159,932	41,398,149,732	43.78	96.7	91.86
E4-5	231,516,610	94.07	246,112,326	37,162,961,226	48.64	96.15	91.13
E4-10	259,464,672	93.85	276,460,032	41,745,464,832	50.04	96.17	91.13
E4-4	210,480,166	95.19	221,104,272	33,386,745,072	43.97	96.7	91.85
E4-8	246,840,606	93.6	263,714,986	39,820,962,886	50.88	96.08	91.22
E4-3	319,840,890	91.42	349,873,104	52,830,838,704	56.64	95.15	89.95

Table S5. Mapping rate and sequencing coverage of whole genome resequencing

Experimental unit	Total_reads	Mapped_reads	Coverage_at_least_4X	Coverage_at_least_10X	Coverage_at_least_20X
E3-1	226,108,071	224,196,897	97.86%	84.08%	38.21%
E3-3	229,372,379	228,384,767	98.49%	93.83%	56.66%
E3-8	251,224,892	249,769,090	98.65%	94.73%	65.77%
E3-9	285,199,690	282,281,915	78.55%	57.02%	38.67%
E3-11	261,681,013	260,404,813	98.62%	95.01%	67.78%
E4-3	322,395,192	317,059,896	54.85%	38.60%	28.80%
E4-4	211,226,491	210,217,001	98.30%	91.40%	44.48%
E4-5	232,698,628	230,931,642	80.41%	60.89%	41.70%
E4-8	248,322,406	245,956,259	87.34%	56.17%	36.53%
E4-10	260,758,700	258,668,988	94.58%	66.65%	34.68%

Table S6. Type and number of INDEL in WGS

Type	Number	Percentage
intronic	813	55.25%
intergenic	581	39.53%
UTR3	26	1.77%
exonic	19	1.26%
downstream	11	0.75%
upstream	10	0.67%
UTR5	6	0.43%
Upstream/downstream	2	0.14%
UTR5/UTR3	2	0.14%
splicing	1	0.07%
total	1471	100.00%

Table S7. Type and number of CNV in WGS

Experimental unit	deletion	duplication	Count of CNV
E3-1	153	3939	4092
E3-3	428	910	1338
E3-8	400	1000	1400
E3-9	0	33185	33185
E3-11	418	968	1386
E4-3	0	30764	30764
E4-4	314	951	1265
E4-5	0	22814	22814
E4-8	0	13642	13642
E4-10	86	11105	11191

Table S8. Type and number of SV in WGS

Experimental unit	CTX	DEL	INS	INV	ITX	Count of SV
E3-1	578	4706	0	1081	6450	12815
E3-3	426	3826	1037	898	4117	10304
E3-8	444	4286	1505	1150	4490	11875
E3-9	428	5918	0	1404	7841	15591
E3-11	389	4449	2024	959	4541	12362
E4-3	452	6525	0	1598	9052	17627
E4-4	374	3551	1440	789	4117	10271
E4-5	550	4817	2325	1401	6029	15122
E4-8	466	5532	3299	1328	6847	17472
E4-10	441	5208	4019	1308	6601	17577

Table S9. SNPs in exon

ID	Site	Chr	Region	HG	LG
ENSGALG00010006948	1812561	1	exonic	A	G
ENSGALG00010005317	2638935	1	exonic	A	C
ENSGALG00010005409	4234452	1	exonic	A	G
ENSGALG00010012147	68465413	1	exonic	A	G
ENSGALG00010012147	68465425	1	exonic	A	G
ENSGALG00010012147	68465428	1	exonic	A	G
ENSGALG00010002194	125190505	1	exonic	A	G
ENSGALG00010002194	125213419	1	exonic	G	A
ENSGALG00010002194	125214994	1	exonic	G	A
ENSGALG00010002800	130476159	1	exonic	G	A
ENSGALG00010006516	172951696	1	exonic	C	T
ENSGALG00010027175	351798	2	exonic	C	T
ENSGALG00010005516	10278676	2	exonic	T	C
ENSGALG00010005516	10279687	2	exonic	T	C
ENSGALG00010005485	11427307	2	exonic	A	G
ENSGALG00010005757	14256056	2	exonic	T	C
ENSGALG00010005299	15520083	2	exonic	A	C
ENSGALG00010011022	68893981	2	exonic	T	C
ENSGALG00010001749	97972667	2	exonic	A	G
ENSGALG00010002357	98013854	2	exonic	G	A
ENSGALG00010005542	103935424	2	exonic	T	C
ENSGALG00010017563	8634643	3	exonic	T	C
ENSGALG00010002758	97048899	3	exonic	C	T
ENSGALG00010002758	97054550	3	exonic	T	C
ENSGALG00010002758	97071216	3	exonic	A	C
ENSGALG00010002832	97133197	3	exonic	C	T
ENSGALG00010002832	97157097	3	exonic	C	T
ENSGALG00010003560	47712941	4	exonic	G	C
ENSGALG00010003560	47713059	4	exonic	C	A
ENSGALG00010004237	49570910	4	exonic	A	G
ENSGALG00010013459	66282790	4	exonic	A	G
ENSGALG00010013459	66301234	4	exonic	T	C
ENSGALG00010015353	69359918	4	exonic	C	T
ENSGALG00010014681	69703019	4	exonic	G	C
ENSGALG00010016229	81438178	4	exonic	T	C
ENSGALG00010016229	81438298	4	exonic	A	G
ENSGALG00010016229	81438310	4	exonic	A	G
ENSGALG00010016229	81438440	4	exonic	G	A
ENSGALG00010016263	81448048	4	exonic	T	C
ENSGALG00010016369	81615803	4	exonic	T	C
ENSGALG00010016385	81677885	4	exonic	G	T

ENSGALG00010017619	82411757	4	exonic	C	A
ENSGALG00010017619	82411758	4	exonic	T	C
ENSGALG00010017619	82411778	4	exonic	A	G
ENSGALG00010017619	82411781	4	exonic	A	G
ENSGALG00010017619	82411820	4	exonic	T	C
ENSGALG00010017619	82424083	4	exonic	T	A
ENSGALG00010021114	8848306	5	exonic	G	A
ENSGALG00010020629	9430987	5	exonic	G	T
ENSGALG00010023209	11382433	5	exonic	A	G
ENSGALG00010023238	11454396	5	exonic	T	C
ENSGALG00010025330	11567478	5	exonic	G	A
ENSGALG00010024347	11671868	5	exonic	T	G
ENSGALG00010024347	11672574	5	exonic	A	C
ENSGALG00010024347	11672599	5	exonic	A	C
ENSGALG00010024347	11672639	5	exonic	A	G
ENSGALG00010024347	11672641	5	exonic	G	A
ENSGALG00010024347	11672655	5	exonic	A	G
ENSGALG00010024347	11672680	5	exonic	A	T
ENSGALG00010024347	11672761	5	exonic	G	A
ENSGALG00010024347	11672785	5	exonic	C	T
ENSGALG00010024347	11675137	5	exonic	G	T
ENSGALG00010024347	11682997	5	exonic	A	G
ENSGALG00010024161	13146367	5	exonic	G	A
ENSGALG00010025386	16676017	5	exonic	G	C
ENSGALG00010020416	24917726	5	exonic	G	A
ENSGALG00010019875	28343645	5	exonic	A	G
ENSGALG00010019875	28343743	5	exonic	G	A
ENSGALG00010019875	28343925	5	exonic	G	A
ENSGALG00010019875	28344081	5	exonic	C	G
ENSGALG00010019875	28344329	5	exonic	A	T
ENSGALG00010019875	28344433	5	exonic	C	T
ENSGALG00010019875	28367748	5	exonic	A	G
ENSGALG00010019875	28368175	5	exonic	G	A
ENSGALG00010019931	28416219	5	exonic	C	T
ENSGALG00010019931	28425532	5	exonic	G	A
ENSGALG00010019931	28426765	5	exonic	T	C
ENSGALG00010019931	28426793	5	exonic	C	T
ENSGALG00010019931	28431161	5	exonic	G	A
ENSGALG00010019931	28431256	5	exonic	T	C
ENSGALG00010020008	28477189	5	exonic	G	A
ENSGALG00010020096	28574569	5	exonic	C	A
ENSGALG00010023319	28995931	5	exonic	A	G
ENSGALG00010023319	28998839	5	exonic	G	A
ENSGALG00010023319	29001752	5	exonic	G	C

ENSGALG00010023319	29005216	5	exonic	C	T
ENSGALG00010023319	29024336	5	exonic	G	C
ENSGALG00010004553	37790777	5	exonic	C	T
ENSGALG00010005107	37870320	5	exonic	C	T
ENSGALG00010005146	37972296	5	exonic	T	C
ENSGALG00010017687	43530321	5	exonic	C	T
ENSGALG00010017687	43595548	5	exonic	C	T
ENSGALG00010017776	43747740	5	exonic	A	G
ENSGALG00010017098	54381740	5	exonic	A	G
ENSGALG00010020524	54896869	5	exonic	T	C
ENSGALG00010020524	54900811	5	exonic	A	G
ENSGALG00010020524	54900820	5	exonic	A	G
ENSGALG00010020524	54900829	5	exonic	A	G
ENSGALG00010020524	54900883	5	exonic	G	A
ENSGALG00010021240	24902426	6	exonic	G	A
ENSGALG00010022310	28343551	6	exonic	C	A
ENSGALG00010025836	12154757	7	exonic	C	T
ENSGALG00010025984	12204781	7	exonic	C	T
ENSGALG00010021734	12591207	7	exonic	A	G
ENSGALG00010010334	2594773	8	exonic	C	T
ENSGALG00010010334	2595322	8	exonic	G	A
ENSGALG00010010334	2595367	8	exonic	A	G
ENSGALG00010010358	2642750	8	exonic	A	G
ENSGALG00010012498	2790215	8	exonic	T	C
ENSGALG00010026128	20839590	8	exonic	G	A
ENSGALG00010026128	20839596	8	exonic	C	A
ENSGALG00010026128	20839605	8	exonic	G	A
ENSGALG00010026581	9640492	9	exonic	G	A
ENSGALG00010026581	9669872	9	exonic	A	G
ENSGALG00010001253	20381089	9	exonic	G	T
ENSGALG00010001267	20674988	9	exonic	C	T
ENSGALG00010021051	8538051	14	exonic	C	T
ENSGALG00010021058	8545581	14	exonic	G	A
ENSGALG00010021058	8546300	14	exonic	A	G
ENSGALG00010021058	8546636	14	exonic	G	A
ENSGALG00010021058	8546910	14	exonic	A	G
ENSGALG00010021058	8547098	14	exonic	T	C
ENSGALG00010017809	8578173	14	exonic	T	G
ENSGALG00010017822	8591600	14	exonic	A	G
ENSGALG00010017822	8591612	14	exonic	T	C
ENSGALG00010018922	8661541	14	exonic	A	G
ENSGALG00010018922	8674530	14	exonic	C	T
ENSGALG00010018922	8674889	14	exonic	A	G
ENSGALG00010018922	8679724	14	exonic	G	A

ENSGALG00010018948	8689269	14	exonic	A	G
ENSGALG00010018948	8702833	14	exonic	G	A
ENSGALG00010018948	8703108	14	exonic	A	G
ENSGALG00010018948	8703121	14	exonic	C	T
ENSGALG00010018948	8703148	14	exonic	G	A
ENSGALG00010019005	8728435	14	exonic	C	T
ENSGALG00010019005	8728732	14	exonic	T	C
ENSGALG00010019005	8728744	14	exonic	T	C
ENSGALG00010019005	8728954	14	exonic	G	A
ENSGALG00010019043	8750410	14	exonic	T	C
ENSGALG00010019051	8757741	14	exonic	G	A
ENSGALG00010019051	8759392	14	exonic	G	A
ENSGALG00010019083	8766935	14	exonic	T	A
ENSGALG00010019083	8790380	14	exonic	G	A
ENSGALG00010019120	8816903	14	exonic	T	C
ENSGALG00010019120	8816904	14	exonic	T	C
ENSGALG00010019120	8817806	14	exonic	G	A
ENSGALG00010019120	8818309	14	exonic	T	C
ENSGALG00010019120	8821937	14	exonic	G	A
ENSGALG00010026643	8828756	15	exonic	A	G
ENSGALG00010026643	8828918	15	exonic	C	G
ENSGALG00010028561	5887461	17	exonic	G	A
ENSGALG00010028565	5908223	17	exonic	G	A
ENSGALG00010028501	6332041	17	exonic	C	T
ENSGALG00010029436	4024350	18	exonic	A	G
ENSGALG00010029734	4714503	18	exonic	A	C
ENSGALG00010029737	5195848	18	exonic	G	A
ENSGALG00010029409	8538051	18	exonic	G	A
ENSGALG00010029417	8560789	18	exonic	A	G
ENSGALG00010029417	8576357	18	exonic	G	A
ENSGALG00010029545	9521957	19	exonic	C	T
ENSGALG00010029564	9774547	19	exonic	T	C
ENSGALG00010029566	9784655	19	exonic	G	A
ENSGALG00010029574	9837819	19	exonic	A	G
ENSGALG00010023948	6313893	20	exonic	T	A
ENSGALG00010015516	2645922	23	exonic	A	G
ENSGALG00010015516	2651624	23	exonic	C	T
ENSGALG00010015914	2892421	23	exonic	A	G
ENSGALG00010015914	2900601	23	exonic	C	T
ENSGALG00010016394	3573757	23	exonic	A	G
ENSGALG00010013541	3620283	23	exonic	C	T
ENSGALG00010016600	4103201	23	exonic	A	G
ENSGALG00010016600	4103204	23	exonic	G	A
ENSGALG00010017415	5497110	23	exonic	C	T

ENSGALG00010017373	5565614	23	exonic	T	C
ENSGALG00010004995	245564	31	exonic	G	A
ENSGALG00010024736	1338244	34	exonic	C	T
ENSGALG00010004034	505577	35	exonic	G	A
ENSGALG00010004034	506704	35	exonic	C	T
ENSGALG00010004034	506810	35	exonic	C	T
ENSGALG00010008830	516522	38	exonic	C	T
ENSGALG00010010165	293536	Z	exonic	G	A
ENSGALG00010010165	293543	Z	exonic	G	A
ENSGALG00010000613	28216	MU179273.1	exonic	G	A
ENSGALG00010001800	180738	MU179263.1	exonic	G	A
ENSGALG00010000414	4641	JAENSK010000034.1	exonic	G	A
ENSGALG00010000402	10751	JAENSK010000592.1	exonic	C	T

Table S10. SNPs in UTR5

ID	Site	Chr	Region	HG	LG
ENSGALG00010009600	13075339	1	UTR5	C	T
ENSGALG00010022478	79254780	1	UTR5	T	C
ENSGALG00010002142	125160447	1	UTR5	A	C
ENSGALG00010002142	125160475	1	UTR5	C	A
ENSGALG00010002142	125161255	1	UTR5	G	T
ENSGALG00010002142	125161307	1	UTR5	C	T
ENSGALG00010002142	125161378	1	UTR5	T	C
ENSGALG00010002142	125161575	1	UTR5	T	C
ENSGALG00010002142	125161683	1	UTR5	C	T
ENSGALG00010003261	119211802	2	UTR5	T	C
ENSGALG00010003261	119219527	2	UTR5	T	C
ENSGALG00010003261	119219611	2	UTR5	T	C
ENSGALG00010003261	119219689	2	UTR5	G	T
ENSGALG00010003261	119220193	2	UTR5	C	T
ENSGALG00010003261	119220488	2	UTR5	A	T
ENSGALG00010003261	119223138	2	UTR5	A	G
ENSGALG00010003261	119223780	2	UTR5	G	A
ENSGALG00010003261	119223853	2	UTR5	C	T
ENSGALG00010003261	119224304	2	UTR5	A	G
ENSGALG00010003261	119224440	2	UTR5	C	T
ENSGALG00010003261	119224531	2	UTR5	G	A
ENSGALG00010003261	119226394	2	UTR5	C	A
ENSGALG00010003261	119226595	2	UTR5	T	A
ENSGALG00010003261	119227911	2	UTR5	C	T
ENSGALG00010003261	119229923	2	UTR5	A	G
ENSGALG00010009344	135586268	2	UTR5	C	A

ENSGALG00010019243	10031395	3	UTR5	G	C
ENSGALG00010015989	17006408	3	UTR5	A	G
ENSGALG00010001896	90629894	3	UTR5	C	T
ENSGALG00010003348	95956345	3	UTR5	A	G
ENSGALG00010003348	95956353	3	UTR5	T	C
ENSGALG00010002832	97120896	3	UTR5	T	C
ENSGALG00010014632	69436604	4	UTR5	C	T
ENSGALG00010014632	69454728	4	UTR5	T	C
ENSGALG00010014632	69454878	4	UTR5	T	C
ENSGALG00010025330	11579089	5	UTR5	C	T
ENSGALG00010025580	15519168	5	UTR5	A	G
ENSGALG00010019875	28329653	5	UTR5	C	T
ENSGALG00010019998	28471806	5	UTR5	C	T
ENSGALG00010020015	28487384	5	UTR5	T	C
ENSGALG00010011212	2327710	7	UTR5	C	T
ENSGALG00010025960	12174799	7	UTR5	C	T
ENSGALG00010025960	12174959	7	UTR5	G	A
ENSGALG00010023473	4746634	9	UTR5	A	G
ENSGALG00010023202	9581666	9	UTR5	C	T
ENSGALG00010023274	9813957	9	UTR5	T	C
ENSGALG00010009117	8693522	13	UTR5	A	G
ENSGALG00010017809	8577940	14	UTR5	T	C
ENSGALG00010017822	8590822	14	UTR5	C	T
ENSGALG00010018922	8661278	14	UTR5	C	T
ENSGALG00010019005	8725992	14	UTR5	A	G
ENSGALG00010019083	8764918	14	UTR5	C	T
ENSGALG00010019083	8766730	14	UTR5	C	A
ENSGALG00010019083	8766851	14	UTR5	C	G
ENSGALG00010028618	6086495	17	UTR5	A	G
ENSGALG00010028232	8697757	17	UTR5	T	C
ENSGALG00010029312	7575736	18	UTR5	A	C
ENSGALG00010029171	1337295	19	UTR5	T	C
ENSGALG00010015914	2903124	23	UTR5	G	C
ENSGALG00010015914	2903304	23	UTR5	G	T
ENSGALG00010015914	2903502	23	UTR5	T	C
ENSGALG00010025423	4069359	26	UTR5	A	G
ENSGALG00010025423	4070415	26	UTR5	C	T

Table S11. Group specific SNP sites located within genes

Gene	Pos	Chr	HG	LG
ALK	8634643	3	T	C
ATP10D	66282790	4	A	G
BATF	37790777	5	C	T
BCHE	20674988	9	C	T
C14H16ORF52	8578173	14	T	G
C2H5ORF22	68893981	2	T	C
CCP110	8545581	14	G	A
	8546910	14	A	G
CHCHD3	2638935	1	A	C
DAGLB	8816903	14	T	C
	8816903	14	T	C
DCLRE1A	28343551	6	C	A
DTHD1	69703019	4	G	C
EEF2K	8661541	14	A	G
ENSGALG00010000613	28216	MU179273.1	G	A
ENSGALG00010001253	20381089	9	G	T
ENSGALG00010010165	293536	Z	G	A
	293543	Z	G	A
ENSGALG00010016229	81438178	4	T	C
	81438310	4	A	G
	81438178	4	T	C
	81438310	4	A	G
ENSGALG00010020524	54896869	5	T	C
ENSGALG00010024161	13146367	5	G	A
ENSGALG00010029737	5195848	18	G	A
LARP4B	10278676	2	T	C
	10279687	2	T	C
LPIN1	97133197	3	C	T
MSANTD1	81615803	4	T	C
	81615803	4	T	C
NEDD8	506704	35	C	T
	506810	35	C	T
ODAD2	15520083	2	A	C
OTOA	8790380	14	G	A
	8790380	14	G	A
	8766935	14	T	A
OTOG	11671868	5	T	G
	11672599	5	A	C
	11672639	5	A	G
	11672641	5	G	A
	11672680	5	A	T

	11672761	5	G	A
	11672785	5	C	T
	11675137	5	G	T
PLEKHH1	28343645	5	A	G
	28343925	5	G	A
	28344081	5	C	G
	28344329	5	A	T
POLN	82411757	4	C	A
POLR3E	8703108	14	A	G
RAB31	98013854	2	G	A
RP1-27O5.3	5565614	23	T	C
SLK	24902426	6	G	A
TGFB3	37972296	5	T	C
TTC7B	43530321	5	C	T
	43595548	5	C	T
VWA3A	8591600	14	A	G
	8591600	14	A	G
	8591612	14	T	C
ZFYVE26	28416219	5	C	T
	28425532	5	G	A
	28426793	5	C	T
	28431161	5	G	A

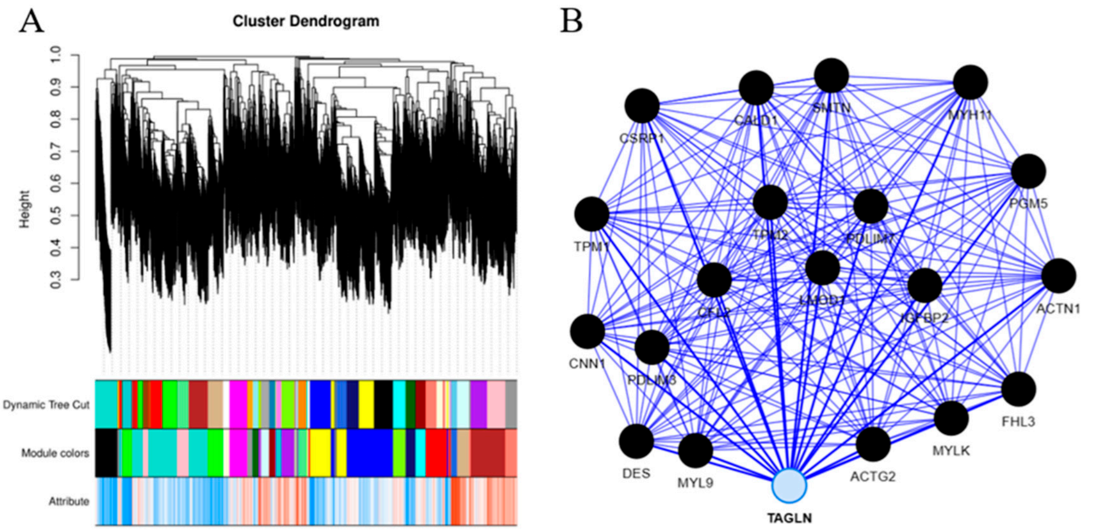

Figure S1. Visual representations of the gene co-expression networks and co-expression network of core genes in key modules. (A) The cluster dendrogram of all genes obtained from all samples. Hierarchical clustering of all genes constructed from RNA-Seq. The colored bars (below) directly correspond to the module (color) designation for the gene clusters. Twenty-three distinct gene modules or groups of genes with high topological overlap were identified. To distinguish between modules, we designated each module with an arbitrary color; grey modules represented poorly connected genes. (B) Hub genes in this highly relevant module.
